# Practice guidelines for outpatient parenteral antimicrobial therapy (OPAT) in Germany

**DOI:** 10.1007/s15010-025-02619-4

**Published:** 2025-08-22

**Authors:** Lukas Tometten, Ulrike Trost, Linda Jürgens, Stephan Achterberg, Lukas Arenz, Franz Audebert, Markus Bickel, Sebastian Dolff, Rika Draenert, Silke Ewering, Julia Fischer, Anette Friedrichs, Stefan Hagel, Annette Hennigs, Dagmar Horn, Caroline Isner, Elham Khatamzas, Christian Lanckohr, Henriette Lang, Hanna Matthews, Beate Sigrid Müller, Jennifer Neubert, Stefan Schmiedel, Arne Simon, Phil-Robin Tepasse, Frederike Waldeck, Clara Lehmann, Miriam Stegemann

**Affiliations:** 1 Department I of Internal Medicine, Division of Infectious Diseases , Medical Faculty and University Hospital Cologne, University of Cologne , Kerpener Str. 62, 50937 Cologne, Germany; 2 German Center for Infection Research, Partner Site Bonn-Cologne, Cologne, Germany; 3 Department of Infectious Diseases, Respiratory Medicine and Critical Care, Freie Universität Berlin and Humboldt-Universität zu Berlin, Berlin Institute of Health, Charité - University Medicine Berlin, Berlin, Germany; 4 Department of Infectious Diseases, Vivantes Klinikum, Berlin, Germany; 5 Antibiotic Stewardship Programme, LMU Klinikum, Munich, Germany; 6 Praxiszentrum Alte Mälzerei, Regensburg, Germany; 7 Infektiologikum Frankfurt, Frankfurt, Germany; 8 Department of Infectious Diseases, West German Centre of Infectious Diseases, Essen University Hospital, Essen, Germany; 9 Department for Internal Medicine I, University Hospital Schleswig-Holstein, Campus Kiel, Kiel, Germany; 10 Faculty of Medicine, Center for Molecular Medicine Cologne (CMMC), University Hospital of Cologne, University of Cologne, Cologne, Germany; 11 Department of Internal Medicine B for Gastroenterology, Hepatology, Endocrinology and Clinical Infectiology, University Hospital Münster, Münster, Germany; 12 Institute for Infectious Diseases and Infection Control, Jena University Hospital, Jena, Germany; 13 Section Infectious Diseases, I. Medical Department, University Medical Center Hamburg-Eppendorf, Hamburg, Germany; 14 Department of Infectious Diseases and Tropical Medicine, Center for Infectious Diseases, University Hospital Heidelberg, Heidelberg, Germany; 15 Partner site Heidelberg, German Center for Infection Research, Heidelberg, Germany; 16 Antibiotic Stewardship Team, Institute of Hygiene, University Hospital Münster, Münster, Germany; 17 Outpatient Center for Infectious Diseases, University Medical Center Hamburg-Eppendorf, Hamburg, Germany; 18 Faculty of Medicine and University Hospital Cologne, Institute of General Practice, University of Cologne, Cologne, Germany; 19 KIJU Praxis Neuss, Praxis für Kinder- und Jugendmedizin, Neuss, Germany; 20 Paediatric Haematology and Oncology, Universitätsklinikum des Saarlandes, Homburg, Germany; 21 Infectious Disease Clinic, University Hospital Schleswig-Holstein, Campus Luebeck, Luebeck, Germany

**Keywords:** Outpatient parenteral antimicrobial therapy (OPAT), Antimicrobial stewardship (AMS), Antimicrobial treatment (AMT), Guideline, Infectious diseases (ID)

## Abstract

**Purpose:**

The practice guideline for outpatient parenteral antimicrobial therapy (OPAT) aims to encourage broader adoption of OPAT into routine clinical practice in Germany.

**Methods:**

The guideline was developed according to the guideline development framework by the Association of the Scientific Medical Societies (AWMF) in Germany. Literature search was conducted, and expert recommendations were formulated through consensus and published as an AWMF S1 guideline (expert group recommendations with consensus development in an informal process).

**Results:**

OPAT is a safe and effective alternative to inpatient care for managing selected infectious diseases (ID) entities, which require intravenous antimicrobial therapy (AMT). ID specialists play a critical role in determining the indications for OPAT, the selection of suitable patients and the development of treatment plans. Specialist-led OPAT programs have been shown to enhance treatment efficacy, reduce hospital readmissions, and decrease healthcare costs. A structured, checklist-based approach is used to evaluate infection severity, available therapeutic options, patient comorbidities, and home care conditions. Adherence to antimicrobial stewardship (AMS) principles as well as regular clinical and laboratory monitoring are essential to ensure appropriate antimicrobial use and minimize adverse events, catheter-related complications and the risk of resistance. The selection of adequate vascular access is based on patient-specific factors, characteristics of the indicated antimicrobial and treatment duration, optimizing both safety and patient comfort.

**Conclusion:**

OPAT is a safe, cost-effective alternative to inpatient care, requiring specialists’ ID expertise and AMS. The guideline provides a framework for successful implementation in Germany.

## Background

**Fig. 1:**
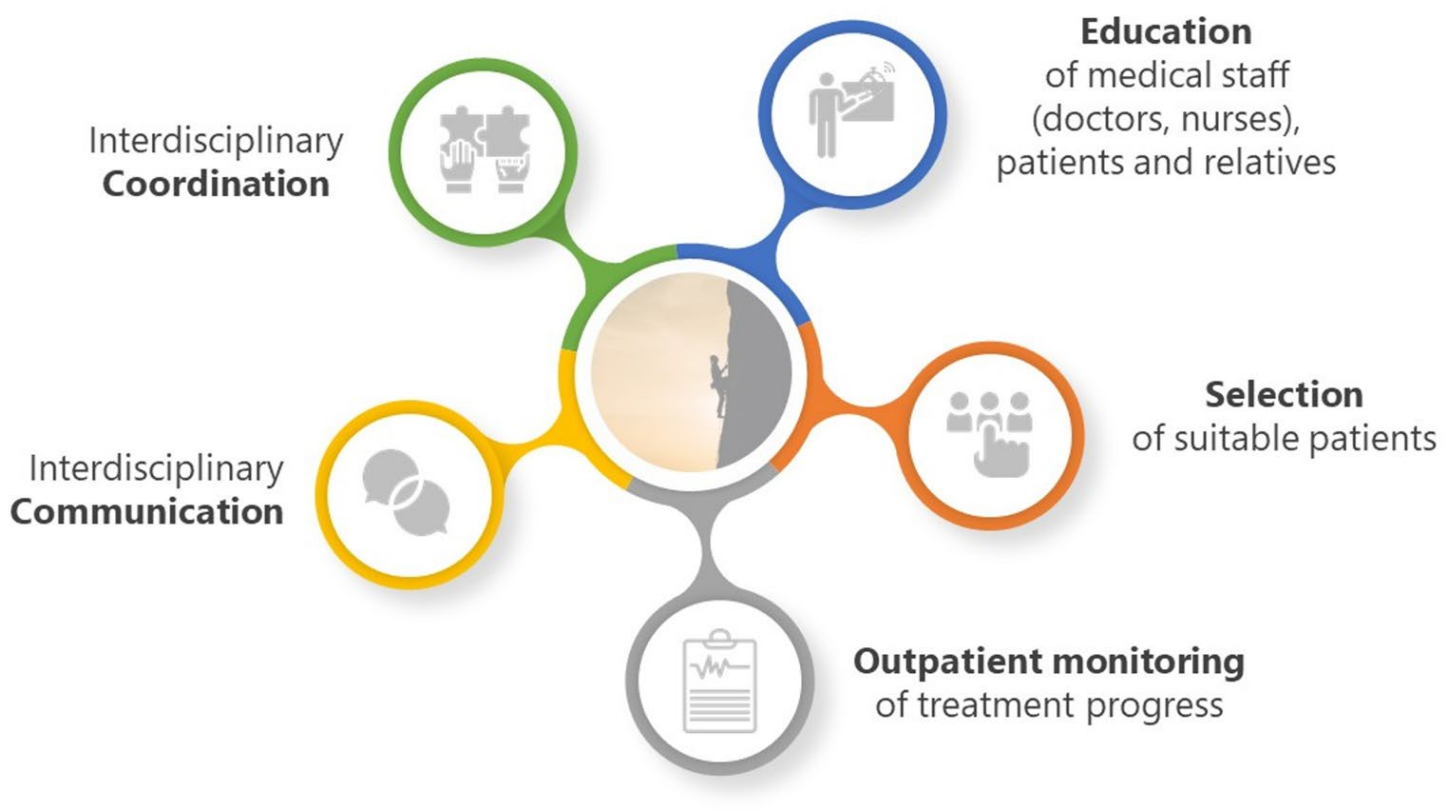
Challenges of OPAT

Outpatient parenteral antimicrobial therapy (OPAT) refers to the intravenous administration of antimicrobial agents outside of an inpatient setting, including application at home, in outpatient clinics, medical practices, and long-term care facilities. It is primarily used for patients who require antimicrobial therapy (AMT) when oral treatment alternatives remain unsuitable. Many countries have established OPAT programs, ensuring structured and effective provision of care. Extensive evidence supports OPAT’s safety and efficacy as a viable alternative to inpatient care. A study published in 2022, which assessed OPAT availability across 30 European countries, outlined the recommended composition of specialized OPAT teams including clinicians, infectious disease (ID) specialists, clinical pharmacists and collaborating closely with outpatient nursing care providers and general practitioners (GP). This cooperative approach effectively addresses the complex, interdisciplinary challenges associated with OPAT, ensuring optimal patient care (see Fig. 1) [1–8].

In principle, OPAT can be utilized for any infection requiring parenteral AMT without oral treatment options. Its feasibility depends on patient characteristics including clinical stability, the severity of the infection and the availability of the necessary infrastructure with an OPAT team. OPAT candidates can be identified in both inpatient and outpatient clinics or emergency care settings, in both pediatric and adult populations without age restriction. OPAT-treated elderly patients, including those with comorbidities, have demonstrated mortality rates comparable to those receiving inpatient care, as well as similar readmission rates to younger adults treated with OPAT [9–11]. Common infections treated with OPAT include endocarditis, osteomyelitis, septic arthritis, skin and soft tissue infections, device related infections, central nervous system infections, and infections caused by resistant or difficult-to-treat pathogens [2, 12]. The increasing prevalence of complex ID with extended therapy durations has led to an increased need for OPAT [1, 13, 14].

OPAT outcomes are assessed based on infection resolution, patient safety, and satisfaction, the latter is generally reported to be high [15]. Complications such as vascular access associated infections (ranging from 2.3 to 9%) are uncommon [15–17]. Treatment failures, e.g. in the context of antimicrobial resistance (AMR) or inadequate source control, underscore the importance of regular monitoring and prompt diagnostic assessment in the outpatient setting. However, treatment failure in OPAT patients is rare when OPAT is managed by an OPAT team with ID specialists, emphasizing the importance of expert oversight and availability of timely interventions to optimize appropriate management and minimize unintended consequences of AMT.

The benefits of OPAT include a shortened hospital stay, resulting in lower direct hospital and staffing costs, as well as indirect costs, such as reduced employee absenteeism [18–21]. Health-economic analyses, as well as data from the UK OPAT registry study show that OPAT, regardless of the indication or administration form, is associated with significant cost savings [22, 23]. A systematic analysis of the cost-effectiveness of OPAT found that the costs of inpatient parenteral anti-infective therapy were 1.1 to 17.34 times higher than with OPAT [24]. The K-APAT study conducted in Germany showed that OPAT resulted in a total reduction of 1,782 inpatient hospital days in 78 patients [25].

Germany´s universal healthcare system generally provides equitable access to care and antimicrobials for all residents, regardless of income or social status. In Germany, both self-administration (S-OPAT) and visiting nurse services (H-OPAT) are viable options for OPAT, and the best approach depends on individual patient needs and circumstances. Nevertheless, to date, OPAT is rarely a routine part of patient care in Germany. The OPAT guideline representing expert recommendations developed through informal consensus aims to provide a structured framework to address this gap and encourage broader adoption of OPAT with the availability of OPAT teams into routine clinical care in Germany.

## Methods

The practice guideline “Outpatient Parenteral Antibiotic Therapy (OPAT)” was developed according to the guideline development framework of the Association of the Scientific Medical Societies in Germany (AWMF) (https://www.awmf.org/regelwerk). The guideline development group (GDG) consists of representatives from selected hospitals in Germany that routinely offer OPAT, from the outpatient sector with OPAT experience, additional OPAT-experienced physicians, pharmacists, scientists, and representatives from the German Society for Infectious Diseases (DGI) as leading medical society, and five further medical societies: the German Society for Internal Medicine (DGIM), the German Society for Pediatric Infectious Diseases (DGPI), the Paul-Ehrlich-Society for Infection Therapy (PEG), the German Society of General Practice and Family Medicine (DEGAM), the Federal Association of German Hospital Pharmacists (ADKA)) and the German Association of Outpatient Physicians for Infectious Diseases and HIV Medicine (dagnä). The declaration of interest management of the GDG including submission, review and clearance was performed according to the AWMF framework (https://www.awmf.org/regelwerk).

The development of clinical questions and formulation of recommendations followed an unstructured literature search which was conducted through “Medline” and the “Cochrane Register” without language restrictions. Relevant documents and publications were identified and evaluated. The evidence base for each recommendation is indicated in the relevant chapter of the guideline. Consensus among the GDG for the recommendations was reached in multiple online meetings and through the circulation of the draft. The strength of the consensus was classified in four categories: strong consensus (> 95% of participants), consensus (> 75–95% of participants), majority agreement (> 50–75% of participants), and no consensus (≤ 50% of participants). The final document was approved by the board of the DGI as leading medical society and the boards of all collaborating medical societies. The recommendations presented here were classified as “expert group recommendations” with “consensus development in an informal process” and the guideline was published as a S1 guideline according to the AWMF guideline classification.

The addendum to the guideline contains a statement on aspects on OPAT in pediatric populations in Germany. The statement including recommendations can be found in a separate publication [26].

The guideline was developed without specific funding or financial support.

Here, we present and discuss a selection of key recommendations. A summary with the full set of the guideline recommendations can be found in Table 1.

**Table 1:** Practice guidelines for outpatient parenteral antimicrobial therapy (OPAT) in Germany - recommendations (full list)

Definition of OPAT, Indication, Setting, and Outpatient Therapy Management			
1	How is OPAT defined?	OPAT refers to the parenteral administration of AMT outside the inpatient setting. This includes the administration of antimicrobials athome, in outpatient clinics, physician offices, or long-term care facilities.	Strong consensus (100% agreement)
2	Who is responsible for setting the indication?	Identification of OPAT patients and the development of the treatment plan by an ID specialist is recommended. An ID specialist is defined as a “specialist in internal medicine and infectious diseases” or “clinical specialist with a focus on infectious diseases or additional training in infectious diseases.” (according to the “German Medical Association” (Bundesärztekammer) as public corporations responsible for the medical self-management. Their work is based on the “Chamber Law for Health Care Professions” (Heilberufekammergesetz))	Consensus (84% agreement*)
3	Who holds responsibility for managing outpatient therapy?	OPAT management should be conducted under the guidance of an ID specialist and in collaboration with GP.	Consensus (84% agreement*)
**Which patients are suitable candidates for OPAT?**			
4	How should patients be identified for OPAT?	It is recommended to use a checklist with selection criteria to identify patients with ID who are eligible for OPAT.	Strong consensus (100% agreement)
5	Where should patients be identified for OPAT?	OPAT patients should be identified both in the inpatient and outpatient setting.	Strong consensus (100% agreement)
**OPAT for Special Patient Groups**			
6	Is OPAT a therapy option for elderly people?	The indication for OPAT should also be evaluated for elderly people.	Strong consensus (100% agreement)
7	Is OPAT a therapy option for people who inject drugs?	Patients who inject drugs should not be excluded from OPAT by default.	Strong consensus (100% agreement)
8		The AMT should be administered in an outpatient facility (e.g., substitution outpatient clinic).	Strong consensus (100% agreement)
9		The indication for the placement of a vascular catheter should be made based on very strict criteria and critically questioned to prevent misuse of the intravenous access.	Strong consensus (100% agreement)
10	Is OPAT a therapy option for homeless people?	Homeless people should not be excluded from OPAT by default.	Strong consensus (100% agreement)
11		The administration of antimicrobials should take place in an outpatient facility (e.g., clinic, doctor’s office, etc.).	Strong consensus (100% agreement)
**Vascular Access**			
12	Which type of intravenous access should be chosen?	The type of intravenous access should be selected based on the specific patient characteristics, the selected medication, the available infrastructure for insertion and maintenance, and most importantly, the planned duration of the AMT.	Strong consensus (100% agreement)
13	Who should be responsible for the selection of the vascular catheter?	The selection of the vascular access for OPAT should be made as part of the patient evaluation by the OPAT team.	Strong consensus (100% agreement)
14	How should intravenous accesses be managed during OPAT?	Regular dressing changes should be performed by trained individuals, as indicated by the manufacturer.	Strong consensus (100% agreement)
15		Patients should be examined regularly by their treating physicians to monitor intravenous access.	Strong consensus (100% agreement)
**Infectious Diseases**			
16	For which ID should OPAT be evaluated as a treatment option?	Any ID with an indication for therapy should be treated with OPAT, provided there is no oral therapy option, the patient selection criteria are met, and the infrastructure for outpatient care is available.	Strong consensus (100% agreement)
**Structural Requirements**			
17	Under what structural conditions should OPAT be considered?	Care by multidisciplinary OPAT teams, which work across sectors in both hospital and outpatient settings, should be a prerequisite for the implementation of OPAT.	Strong consensus (100% agreement)
**Professional Requirements and AMS**			
18	Should OPAT therapy be evaluated according to AMS principles?	OPAT should always be conducted in accordance with AMS principles.	Strong consensus (100% agreement)
19	What is the importance of evaluating oral therapy options?	Oral therapy should be preferred over parenteral therapy.	Strong consensus (100% agreement)
**Chemical-Physical Criteria for the Selection of an Anti-infective Agent**			
20	What chemical-physical criteria should be considered when selecting an anti-infective for OPAT?	The chemical-physical criteria to be considered when selecting an AMT for OPAT include: • Correct dosage and dosage intervals of the AMT • Robust stability data of the AMT • Additional physical and chemical properties of the active substance in relation to the infusion system, solvent, concentration, the use of stability-supporting buffers, and temperature • Side effects (adverse drug reactions) • Interactions with concomitant medication	Strong consensus (100% agreement)
**Stability Criteria for Selecting an Anti-infective Agent**			
21	What stability criteria should be used to classify an anti-infective as suitable for OPAT?	An antimicrobial should be classified as suitable for OPAT based on its stability with regard to temperature and time.	Strong consensus (100% agreement)
22		An antimicrobial should be classified as suitable for OPAT based on its stability regarding maximum concentration, solvent, and buffer.	Strong consensus (100% agreement)
**Role of the OPAT Team Regarding the Preparation**,** Storage**,** Transport**,** and Dispensing of Antimicrobials**			
23	What is the role of the OPAT team regarding the preparation, storage, transport, and dispensing of antimicrobials?	Interdisciplinary collaboration within the multi-professional and cross-sector OPAT team should ensure that the processes related to the preparation, storage, transport, and dispensing of antimicrobials for OPAT patients are safe.	Strong consensus (100% agreement)
**Outpatient Treatment Setting**			
24	In which outpatient treatment situations should OPAT be performed?	The selection of the appropriate treatment setting should be made individually for each patient, depending on compliance, the duration and frequency of administration, the substance, patient factors, and the social environment.	Strong consensus (100% agreement)
**Monitoring During OPAT**			
25	Should regular clinical and laboratory checks be performed for patients receiving OPAT?	OPAT patients should be regularly assessed for clinical and laboratory treatment response, tolerability of AMT, and the early detection of complications.	Strong consensus (100% agreement)
26	How frequently should outpatient follow-up examinations be conducted?	Follow-up visits should occur weekly, or more frequently if necessary.	Strong consensus (100% agreement)
27	Who should conduct the outpatient follow-up examinations?	The management of OPAT should be overseen by an ID specialist, in coordinated collaboration with GP, to ensure the safety, efficacy, and continuity of care.	Strong consensus (100% agreement)

*There was a difference in opinion within the guideline development group, as some members expressed concerns about making the presence of a board-certified specialist in internal medicine and infectious diseases—or a specialist with additional training in infectious diseases—a strict requirement for implementing an OPAT program. They proposed that, in settings where no infectious disease (ID) specialist is available, transitional models should be permitted, such as involving an AMS-certified team member, cooperating with a DGI-recognized center, and holding regular joint OPAT case conferences. Infectious diseases, with the recent establishment of a new specialty in Internal Medicine and Infectious Diseases and the availability of additional training options across nearly all medical disciplines, is expected to play a key role in addressing the current structural and economic challenges facing the German healthcare system. Until this capacity is fully established, transitional solutions will remain necessary in many care settings

## Key recommendations

### ID specialist In OPAT management

**OPAT management**

*The management of OPAT should be overseen by an ID specialist*,* in coordinated collaboration with GP*,* to ensure safety*,* efficacy*,* and continuity of care.*

**Strong consensus (100% agreement)**

**OPAT Indication and Treatment Plan**

*Identification of OPAT patients and the development of the treatment plan by an ID specialist is recommended. An ID specialist is defined as a “specialist in internal medicine and infectious diseases” or “clinical specialist with a focus on infectious diseases or additional training in infectious diseases” (according to the “German Medical Association” (Bundesärztekammer) as public corporations responsible for the medical self-management. Their work is based on the “Chamber Law for Health Care Professions”)).*

**Consensus (84% Agreement)**

OPAT is a safe and effective treatment option for various ID and represents an alternative to inpatient treatment. To guarantee efficacy and safety, a high level of ID expertise is required. Both internationally and in Germany, specialized ID departments have the most experience with OPAT. Outpatient care by ID specialists significantly reduces the rate of readmission within the first two weeks post-discharge [27]. Two retrospective studies showed that patients receiving OPAT were significantly more likely to be readmitted if they did not have outpatient follow-up through ID-led OPAT clinics (58.9% vs. 19.7%). This also applied to patients transferred to subacute rehabilitation centers who could not attend outpatient appointments [28, 29]. In addition, a controlled, quasi-experimental study showed that optimization of an ID-led, interdisciplinary OPAT clinic resulted in significantly fewer medication errors [30]. A retrospective analysis of 8,200 patients undergoing OPAT in the United States of America demonstrated that ID led OPAT care was associated with improved treatment outcomes, including reduced rates of emergency department visits and hospital admissions within 30 days. Moreover, ID led OPAT care was linked to cost reductions [8]. Further studies have also highlighted the positive impact of ID consultations prior to discharge, which led to better treatment outcomes, fewer adverse events, and a reduced number of OPAT initiations for patients who were deemed suitable for oral AMT [15, 30–33].

Given the complexity of the underlying illness and the expertise required, the identification of OPAT patients, the decision to initiate OPAT, and the preparation of a treatment plan, should be carried out by an ID specialist. In the German context, this means that a specialist in internal medicine (or pediatrics in pOPAT) and ID or a specialist with a focus on ID should be leading the OPAT team. In hospitals with a lack of ID specialists, established regional ID networks should be consulted and collaborations with nearby specialized ID centers are encouraged. In Germany, the establishment of a separate specialty for internal medicine and ID was approved in May 2021, which will make more trained specialists available in the future [34].

### Selection of OPAT patients

**Checklist for Identifying Eligible OPAT Candidates**

*It is recommended to use a checklist with selection criteria to identify patients with ID who are eligible for OPAT.*

**Strong Consensus (100% Agreement)**

The proposed OPAT patient selection checklist is intended to assist dedicated OPAT teams in enhancing and ensuring the quality of both processes and outcomes. The first step in determining the indication for OPAT includes assessing the need for AMT. Additionally, suitability for OPAT depends on various factors, including the severity of the infection, the course of disease, the site of infection, the identified pathogen(s) and their resistance profiles, comorbidities, and patient compliance. Key considerations in the decision-making process also include the route of administration (parenteral versus oral), the stability and risk of adverse events associated with AMT, the selection of vascular access, and, critically, the outpatient setting and the patient’s setting at home (see Table 2). The checklist is intended to support the OPAT team in decision-making and highlight key questions during the evaluation and implementation of OPAT. The checklist has not yet been validated in an OPAT population and should currently be considered an expert-based best practice tool approved by the GDG.

**Table 2:** OPAT patient selection checklist

Criteria	Yes	No
**AMT according to AMS criteria**		
Is continuation of AMT indicated?		
Is oral administration of the indicated AMT possible?		
**Assessment of the infection**		
Has the diagnosis of the underlying infectious disease been confirmed?		
Has the clinical course of the infection improved?		
Are further invasive interventions (e.g. surgery) necessary?		
Has a causative pathogen been identified and is it responsive to the selected AMT?		
Does the AMT include antimicrobials that require TDM (e.g., aminoglycosides, glycopeptides)?		
**Selection of medication**		
Is the dosage frequency feasible for outpatient administration?		
Is there reliable data on the stability of the selected antimicrobial agent?		
Is therapeutic drug monitoring (TDM) required? If yes, is it possible to be performed in the outpatient setting?		
Are any adverse drug reactions (ADR) to antimicrobials reported in the medical history of the patient?		
**Vascular access for OPAT**		
Is adequate vascular access present (e.g. PICC line, port)?		
**Suitability of the patient and clinical condition**		
Is the general condition stable and sufficient for the outpatient setting (including stable vital signs)?		
Are there relevant comorbidities that could interfere with OPAT?		
Has the patient been adequately informed about the need for parenteral AMT and regular follow-up examinations?		
Is there a risk of substance misuse?		
Does the patient suffer of a mental illness that might affect OPAT (e.g. anxiety)?		
**Home Setting and Outpatient Therapy Feasibility**		
Does the patient live in an environment suitable for OPAT?		
Is reliable telephone communication with the patient possible?		
Are the patient and/or relatives informed, agreeable, and trained regarding the therapy?		
Has the patient been informed and understood the behavioral measures for therapy- or catheter-associated risks?		
Is wound care necessary?		
Is weekly transport to an outpatient facility for monitoring visits possible?		
Has the mode for prescription of OPAT after discharge been defined?		
Does the patient have a GP who can monitor the OPAT in cooperation with the OPAT team?		
Is the GP informed about the OPAT?		

### Antimicrobial stewardship (AMS)

**Adherence to AMS Principles**

*OPAT should always be conducted in accordance with AMS principles.*

**Strong consensus (100% agreement)**

AMS aims to promote the responsible use of AMT, optimize therapeutic efficacy, reduce the risk of adverse drug reactions (ADRs), curb the spread of antimicrobial resistance (AMR), and avoid unnecessary healthcare costs. The integration of AMS principles into antimicrobial treatment is internationally acknowledged and broadly endorsed [35–38]. The following aspects should be considered when planning treatment: diagnosis and documentation of AMT indication, source control, selection of AMT, evaluation of oral therapy options, appropriate dosing, treatment duration, and assessment of therapeutic response. Patients should be actively involved in the decision-making process through the principles of shared decision-making.

OPAT treatment planning should adhere to established national and local clinical guidelines, including recommended diagnostic evaluations to identify the specific diagnosis and causative pathogen(s). The indication for AMT must be clearly documented. When applicable, adequate source control should be achieved before initiating OPAT. AMT selection should be reviewed regularly, with ongoing assessment for potential transition from intravenous to oral or cessation of therapy. In general, identifying the causative pathogen should be prioritized to enable a shift from empirical to targeted treatment. AMT with the narrowest possible spectrum is recommended to minimize selective pressure on pathogens [39]. Unnecessary broad-spectrum therapy for pragmatic reasons (e.g., due to more favorable administration frequency) should be avoided. Patient selection for OPAT in Germany should continue to follow strict criteria, with a strong emphasis on the assumption of high patient compliance. Although there are positive study data supporting the use of broad-spectrum antibiotics when they lead to ease of administration and patient comfort [40], we take a cautious view of this approach and encourage using the narrowest-spectrum antibiotic possible to minimize the risks associated with AMR.

When determining therapeutic options, it is essential to consider clinical guidelines for the underlying infection, local epidemiological data, pathogen resistance patterns, and individual patient factors. The medication regimen prescribed by the OPAT team must adhere to appropriate dosing requirements. Additionally, the identification of potential drug interactions, ADRs, and the implementation of therapeutic drug monitoring (TDM) are key components of therapy planning and management, with ID and AMS trained pharmacists playing a crucial role [39]. The dosage, dosing interval, and duration of infusion should be tailored to the specific AMT selected and the patient’s clinical condition. The duration of therapy, including initiation and ending dates must be determined in advance in accordance with clinical guidelines and appropriately documented. Throughout the course of therapy, regular clinical evaluations should assess the therapeutic response. Options for oral treatment and termination of OPAT should be routinely reviewed and documented during treatment. Therapy should be adjusted according to AMS principles based on the patient’s clinical response and microbiological findings, with a focus on de-escalation when appropriate. In cases of suboptimal therapeutic outcomes or treatment failure, it is necessary to reassess the initial diagnosis and consider potential contributing factors, such as inadequate source control or the emergence of AMR.

**Preference of Oral Therapy**

*Oral therapy should be preferred over parenteral therapy.*

**Strong consensus (100% agreement)**

When evaluating oral therapy options for the treatment of ID, factors such as bioavailability, molecular size, hydrophilic/lipophilic properties, and tissue penetration at the infection site must be considered [12]. Oral therapy should be preferred if active substances with sufficient bioavailability are available that reach effective therapeutic concentrations at the site of infection targeting the causative pathogens. Careful consideration is required when evaluating oral therapeutic options for pathogens with elevated minimum inhibitory concentrations (MICs), to ensure both the safety and efficacy of treatment. The switch from parenteral to oral treatment has been shown to be safe and effective for many entities [41]. Additionally, a pharmaceutical evaluation of potential drug interactions and ADRs should be conducted. The OPAT team is responsible for ensuring that oral therapy options are consistently evaluated during the course of the program [39].

### Selection of antimicrobial agent

**Chemical-Physical and Stability Criteria for Selecting an Antimicrobial Agent**

*The chemical-physical criteria to be considered when selecting an AMT for OPAT include*:

- *Correct dosage and dosage intervals of the AMT*
- *Robust stability data of the AMT*
- *Additional physical and chemical properties of the active substance in relation to the infusion system*,* solvent*,* concentration*,* the use of stability-supporting buffers*,* and temperature*.
- *Side effects (adverse drug reactions)*.
- *Interactions with concomitant medication*.

**Strong consensus (100% agreement)**.

For the evaluation of pharmaceuticals and infusion systems, product information and manufacturer guidelines offer an initial overview of stability testing. Additional information regarding the properties of active ingredients can be found in the stability database www.stabilis.org. The *Extended Stability for Parenteral Drugs Handbook* (7th edition, 2022) by the American Society of Health-System Pharmacists (ASHP) provides a comprehensive summary of stability data in the form of drug monographs, focusing on various infusion systems, concentrations, solvents, and temperatures. Furthermore, the British Society for Antimicrobial Chemotherapy (BSAC) conducts central stability testing programs for various active substances to gather data under OPAT conditions and offer recommendations [42–45]. Jenkins et al. discuss further aspects of stability data related to extended or continuous infusion at both room temperature and “body-like temperatures” [46–49]. Chapman et al. provide an overview of commonly used antimicrobials and their properties in their *Update Good Practice Recommendations for Outpatient Parenteral Antimicrobial Therapy (OPAT) in Adults and Children in the UK* [2].

Currently, there is limited data on the stability of antimicrobials under “real-life” conditions. Ampicillin, Ampicillin/Sulbactam, Ceftazidime, Meropenem, and Imipenem may be unstable both at room temperature and at higher temperatures during extended infusion periods, with degradation occurring after only a few hours [50–53]. In contrast, Piperacillin/Tazobactam, Benzylpenicillin, and Vancomycin remain stable for up to 24 h at room temperature and at temperatures exceeding 30 °C. The clinical significance of these findings remains unclear, as data are currently lacking. The concentration of the drug and the choice of solvent (e.g., 5% glucose or 0.9% sodium chloride) also significantly affect the physicochemical stability of the agent. The incorporation of buffer systems can help stabilize the pH of a solution, thereby enhancing the stability of the active substance.

Table 3 focuses on elastomeric pumps, as they currently are the most commonly used method for S-OPAT in Germany, where pre-prepared infusions make stability data particularly relevant for clinical practice. The table summarizes the results for commonly used antimicrobials in elastomeric pumps within the context of OPAT, highlighting their physicochemical properties and providing guidance for OPAT teams in selecting appropriate therapies.

**Table 3:** Selection of commonly used antimicrobials within the framework of OPAT and their physicochemical properties in elastomeric pumps

Selection of commonly used antimicrobials within the framework of OPAT and their physicochemical properties in elastomeric pumps									
Pharmaceutical*	Dosage interval (in normal renal function)	Solvent	Stability in elastomeric pumps			Stabilization with a buffer	Continuous administration	Significant ADR	Literature
			2–8 °C	25 °C	> 30 °C				
Antibiotics									
Ampicillin	Every 4–6 h	NaCl	72 h	24 h	5 h	X (phosphate)	Yes		[47, 49, 54]
Benzylpenicillin	Every 4–6 h	Ringers acetate	48 h	24 h	24 h	N/A	Yes		[47, 49, 55]
Ceftriaxone	Once daily	G5	7d	24 h	N/A	N/A	No		[47]
Cefepime	Every 8–12 h	NaCl	7d	24 h	12 h	N/A	No	Neurotoxicity	[47, 49]
Ceftazidime	Every 8 h	NaCl	48 h	8 h	N/A	N/A	No		[47]
Cefazolin	Every 8 h	NaCl, G5	72 h	12 h	12 h	N/A	No		[49, 53]
Daptomycin**	Once daily	NaCl	10d	24 h	N/A	N/A	No	Myopathy, eosinophilic pneumonia (in prolonged treatment)	[56]
Flucloxacillin	Every 4–6 h	NaCl	14d	N/A	24 h	X (citrate)	Yes		[42, 49]
Meropenem	Every 8 h	NaCl	48 h	12 h	6 h	N/A	No		[43, 49, 51]
Imipenem	Every 6 h	NaCl	8 h	3 h	N/A	N/A	No		[49, 52]
Piperacillin/Tazobactam	Every 6–8 h	NaCl	13d	24 h	24 h	X (citrate)	Yes		[44, 49]
Vancomycin	Every 12 h	NaCl	13d	24 h	24 h	N/A	Yes	Nephrotoxicity, thrombophlebitis	[49, 57]
Antifungals									
Caspofungin	Once daily	NaCl	48 h	24 h	N/A	N/A	No	Thrombophlebitis	[49]
Antivirals									
Aciclovir	Every 8 h	NaCl	N/A	14d	24 h	N/A	Yes	Nephrotoxicity, neurotoxicity, thrombophlebitis	[58]

Abbreviations: NaCl: sodium chloride 0.9%; G5: glucose 5%; h: hours; d: days; N/A: not specified; *Stability may vary depending on drug, solvent, infusion system and storage conditions; **OPAT only in selected situations, reserve antibiotic

### Selection of intravenous access

**Catheter selection based on patient characteristics and duration of treatment**

*The type of intravenous access should be selected based on the specific patient characteristics*,* the selected medication*,* the available infrastructure for insertion and maintenance*,* and most importantly*,* the planned duration of the AMT.*

**Strong consensus (100% agreement)**

**Fig. 2:**
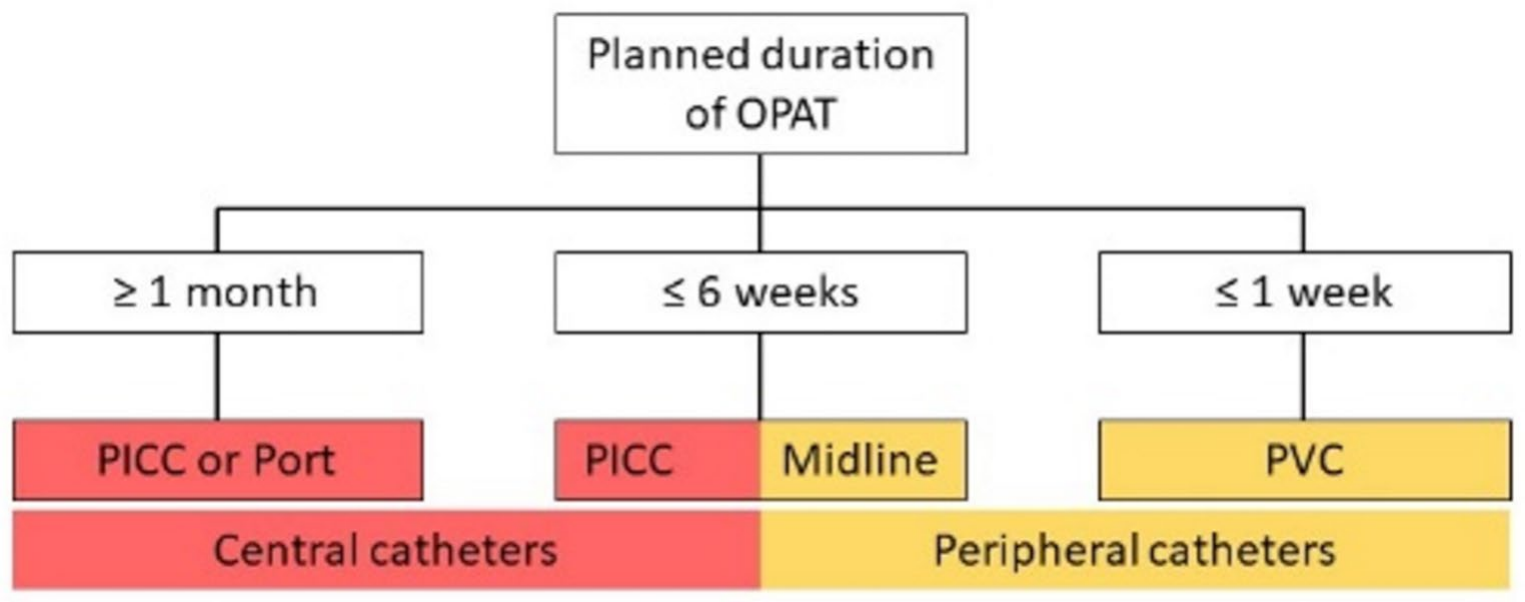
Overview of vascular catheters in relation to OPAT duration (PICC: Peripherally Inserted Central Venous Catheter, PVC: Peripheral Venous Catheter)

The correct selection of the vascular catheter is of particular importance for the administration of OPAT, its management, and the reduction of catheter-associated complications. The decision primarily involves choosing between peripheral (e.g., peripheral venous catheters (PVC), midline catheters) and central venous access (e.g., peripherally inserted central catheter (PICC or PICC line), port catheters), with preference given to the least invasive option with the lowest complication rate for the required duration of treatment. Complication rates are generally low for all catheter types in the context of OPAT [25]. However, complications are comparatively more frequent with peripheral catheters than with central venous catheters (Midline > PICC > Port) [59]. Therefore, catheter selection depends on specific patient characteristics, the medication chosen, and, most importantly, the planned duration of the OPAT. Additionally, the choice of catheter must be adapted to the available infrastructure (e.g. the availability of interventional radiology and/or surgery). Existing access points, such as a port system, which are already in place for other indications, may also be utilized for AMT. Other central venous access devices (e.g., Hickman catheter, Demers catheter) are also suitable [60]. The port system is designed for long-term parenteral therapy (e.g., >1 month) and consists of a subcutaneously implanted chamber and a catheter whose tip is positioned centrally in the superior vena cava. For patients requiring parenteral nutrition or volume therapy, a port catheter is often an appropriate vascular access option. A PICC is suitable for therapies lasting up to 3 months but can also be used longer depending on manufacturer’s guidelines. PICC placement is less time-consuming and less invasive than port implantation, and PICC removal can be performed by medical staff without surgery, whereas a port requires surgical removal. In patients with chronic kidney disease (CKD III-V), PICC placement should be considered with caution, as vascular damage may complicate future creation of an arteriovenous fistula for dialysis. Alternatively, a tunneled central catheter (e.g., Broviac/Hickman) can be used. A midline catheter is another option, with its tip located distal to the axillary vein, classifying it as a peripheral venous catheter. According to manufacturer specifications, midline catheters can remain in place for up to 6 weeks. However, recent evidence suggests that midline catheters may be associated with a higher risk of complications compared to PICCs [61]. Other peripheral venous catheters (PVCs) are generally insufficient for OPAT due to their short dwell time. Figure 2 provides an overview of various intravenous catheter types that are available for OPAT.

### Monitoring

**Regular Monitoring of intravenous access**

*Patients should be examined regularly by their treating physicians to monitor intravenous access.*

**Strong consensus (100% agreement)**

**Regular Dressing Changes**

*Regular dressing changes should be performed by trained individuals*,* as indicated by the manufacturer.*

**Strong consensus (100% agreement)**

Catheter-associated complication rates range from 0 to 6%, or 0.39–0.48 per 1000 catheter days. To reduce the risk of catheter-associated complications in OPAT, patients or their relatives must be thoroughly trained in aseptic techniques of dressing changes and proper catheter flushing, as well as educated on early detection of complications [62]. Furthermore, intravenous access should be regularly examined by trained professionals and patients should be regularly seen by their treating physicians [63]. Finally, patients should also have a 24-hour point of contact for support with a defined safety net in place [2, 64].

Venous thrombosis is one of the most common catheter-associated complications. Risk factors for catheter-associated venous thrombosis in the context of OPAT include the type of intravenous access, duration of OPAT, age, female sex, and the number of anti-infective agents administered [15]. The risk of venous thromboembolism (VTE) in OPAT is not fully understood, and the optimal strategy for thrombosis prophylaxis in patients without other indications for anticoagulation remains unresolved [65]. Currently, there is no general recommendation for initiating thrombosis prophylaxis. In the case of thrombosis during OPAT, anticoagulation should be initiated. If the catheter is in a good position and the clinical situation is tolerable, the catheter may be retained with the use of anticoagulation. Still, the risk of severe bleeding should be considered when deciding whether to retain the catheter [12]. Additionally, a catheter-related infection should always be excluded in the case of thrombosis. If thrombosis occurs, it is recommended to continue anticoagulation for 3 months following catheter removal [66].

**Regular monitoring for treatment response and complications**

*OPAT patients should be regularly assessed for clinical and laboratory treatment response*,* tolerability of AMT*,* and the early detection of complications.*

**Strong consensus (100% agreement)**

**Weekly follow-up visits**

*Follow-up visits should occur weekly*,* or more frequently if necessary.*

**Strong consensus (100% agreement)**

OPAT may lead to ADRs, such as hypersensitivity reactions, blood count changes, gastrointestinal symptoms, antibiotic-associated diarrhea, and *Clostridioides difficile* infections (CDI). Additionally, vascular catheter-associated complications may occur [28, 67, 68]. Clinical monitoring is essential during OPAT to track therapeutic response and address potential complications promptly. In addition to history taking and physical examination, which includes inspection of the catheter insertion site, regular blood tests should be conducted. The unavailability of laboratory parameters during OPAT is an independent risk factor for hospital readmission. In a retrospective cohort study, missing laboratory parameters were associated with a 2.5-fold increased risk of readmission [29]. However, there are insufficient studies to provide evidence-based recommendations regarding the frequency of clinical evaluations and the laboratory parameters to be monitored. A case-control study by Saini et al. showed that readmissions due to OPAT-related complications most commonly occurred within the first two weeks after discharge [27]. Other studies report an increased risk of ADRs with longer durations of parenteral AMT. Briggs et al. noted delayed reactions such as rashes, abdominal pain, leukopenia, thrombocytopenia, and elevated liver enzymes, typically occurring 25 days after the initiation of beta-lactam antibiotic therapy [69]. Eosinophilia, which may develop after the 15th day of therapy, is a predictor of hypersensitivity reactions and/or renal insufficiency [70] and can be anticipated through regular laboratory monitoring. For selected cases, however, the follow-up interval may be individually determined and extended, based on a careful risk–benefit assessment [71]. Regular therapeutic drug monitoring of glycopeptides and aminoglycosides should also be performed to reduce the risk of ADRs and ensure that therapeutic target levels are achieved and toxic drug levels are avoided during OPAT. Moreover, anti-infective therapies are associated with CDI, although a lower incidence of CDI has been observed in OPAT patients compared to hospitalized patients [72]. Changes to the anti-infective therapy should be made in consultation with ID specialists experienced in OPAT management.

In addition to monitoring tolerability, the treatment response to anti-infective therapy should be evaluated through clinical, laboratory, and, if necessary, imaging diagnostics. In a retrospective cohort of 6,120 OPAT patients, more than 94% were successfully treated, with only 5.7% experiencing an infection recurrence [73]. Weekly interdisciplinary meetings within the OPAT team and with involved specialties are recommended for adjusting and discontinuing intravenous anti-infective therapy. At the end of therapy, the end of treatment should be documented, and the vascular access should be removed.

## Conclusion and outlook

OPAT represents a safe and effective treatment option for a range of ID, offering an alternative to inpatient care. Selected patients, for whom no oral AMT option is available can still be safely treated in an outpatient setting. Determining the indication for OPAT and considering AMS principles, are important aspects that must be thoroughly evaluated. This should be done by ID specialists. Furthermore, the success of OPAT also depends on the presence of a specialized multi-professional OPAT team. OPAT offers advantages such as a reduced length of hospital stay, leading to lower direct costs for hospitals and staff, as well as indirect savings through decreased employee absenteeism [18–21]. In addition, limited inpatient care facilities are no longer blocked solely for providing parenteral AMT. Furthermore, OPAT offers substantial potential to strengthen outpatient, cross-sectoral care models. This requires expanding the availability of ID expertise, both in inpatient and outpatient settings, including interdisciplinary, multi-professional OPAT teams that ensure seamless collaboration across sector boundaries. Telemedicine has the potential to play a role in OPAT in the future as it can provide access to ID care in remote areas [74]. While established OPAT programs are part of healthcare systems in many countries worldwide, standardized structures and formal funding models for OPAT in primary and secondary care need to be developed in Germany. This guideline developed through expert consensus constitutes an initial step toward the formal establishment of OPAT as a standard practice in Germany. It aims to provide a structured framework to facilitate the broader implementation of OPAT, including the integration of dedicated OPAT teams, into routine clinical care in Germany.

## Data Availability

No datasets were generated or analysed during the current study.
